# Implementation of a broad public health approach to COVID-19 in Sweden, January 2020 to May 2022

**DOI:** 10.2807/1560-7917.ES.2023.28.41.2300063

**Published:** 2023-10-12

**Authors:** Anders Tegnell, Anna Bessö, Britta Björkholm, Sara Byfors, Johan Carlson, Karin Tegmark Wisell

**Affiliations:** 1 Public Health Agency of Sweden, Solna, Sweden; 2 Public Health Agency of Sweden, Solna, Sweden (until 7 September 2022); 3 Swedish Agency for Health Technology Assessment and Assessment of Social Services (SBU), Stockholm, Sweden (current); 4 Public Health Agency of Sweden, Solna, Sweden (retired as of 1 November, 2021)

**Keywords:** SARS-CoV-2, COVID-19, coronavirus, pandemic, public health, infection control, outbreak, surveillance

## Abstract

In 2020, the world had to adapt to a pandemic caused by a then novel coronavirus. In addition to its direct impact on morbidity and mortality, the COVID-19 pandemic brought unprecedented control measures and challenges to both individuals and society. Sweden has been seen by many as an outlier in the management of the pandemic. It is therefore of special interest to document the actual management of the pandemic in Sweden during its first 2 years and how public health was affected. In the authors opinion, within the Swedish context, it has been possible to achieve a similar level of effect on mortality and morbidity through recommendations as was achieved through stringent legal measures in comparable countries. This is supported by comparisons of excess mortality that have been published. Furthermore, we see in the available data that the consequences on mental health and living habits were very limited for the majority of the population. Trust in public institutions is high in Sweden, which has been important and is part of the context that made it possible to manage a pandemic with relatively ‘soft’ measures. We acknowledge challenges in protecting certain vulnerable groups, particularly during the first and second wave.

## Background

In 2020, the world had to respond to a pandemic caused by a novel coronavirus. In addition to a direct impact in terms of morbidity and mortality, the COVID-19 pandemic brought unprecedented control measures and challenges to both individuals and society.

In this article, we describe how Sweden managed the pandemic, and we illustrate how public health, in a broad sense including also indirect effects on public health by the pandemic and the measures taken, has been affected in the first 2 years of the pandemic. We do not aim to analyse the effects of the different interventions or compare the outcome to that in other countries. For an evaluation of the pandemic in Sweden, we refer the reader to the final report from the Swedish Corona Commission [1].

Sweden, like most countries, initially made substantial efforts to contain the severe acute respiratory syndrome coronavirus 2 (SARS-CoV-2), mainly by testing and contact tracing people arriving from countries with known spread of the virus. The first case was recorded on 30 January and cases were managed for more than a month until there was an established community transmission in large parts of the country in the spring of 2020. Behind the community transmission was a heavy influx of cases during the weeks in February and March, coinciding with school holiday breaks when many Swedes travelled abroad. The Swedish Transport Agency registered more than 1 million individual air travel entries (n = 544,680) and exits (n = 635,338) to and from Sweden between 24 February 2020 and 15 March 2020, a volume several times higher than in the weeks before [2]. Cases infected with SARS-CoV-2 were imported from countries from all over the world, including countries where little was known about the virus spread at that time. This influx rapidly exceeded the available testing and contact tracing capacity and forced a shift in the strategy with the aim to slow down the spread of the virus and to protect identified risk groups, mainly people older than 65 years and people with certain chronic diseases. A broad public health approach became a prerequisite of the adapted strategy. In particular, there was a focus on the health and well-being of children and on vulnerable groups in society, while also aiming to safeguard health equity. With this broad public health approach as a foundation, emphasis was placed on measures that minimised physical contacts in settings with a high risk of disease transmission and that were sustainable over the time the pandemic was expected to continue, and that took into account the potential impact imposed on public health in general.

## The Swedish response to COVID-19 in context

Sweden has three levels of governance: national, regional and local, with 21 regions and 290 municipalities. The principle of local self-governance gives the regions and municipalities the right to design and structure their activities based on local conditions [3]. The regions are, among other things, responsible for ensuring that residents have access to high quality health and care services. The municipalities are, for example, responsible for childcare, primary and secondary education and care for older people.

Sweden’s constitution does not allow the declaration of a state of emergency. Fundamental civil rights and freedoms can only be suspended in the case of war or immediate threat of war. Public health emergencies are therefore regulated by existing laws, which allocate responsibilities for implementing measures in different governing areas.

The Communicable Diseases Act is based on an individual’s own responsibility and voluntary actions, except for individuals with a diagnosis of a dangerous communicable disease who are mandatorily required to follow the advice given by their doctor to avoid spread of the disease. Furthermore, measures taken under the Communicable Diseases Act may not be more far-reaching than what the person taking the decision considers justifiable in regard to the specific danger of the disease to human health (the principle of proportionality).

The Public Health Agency of Sweden (PHAS) is independent from the Government. The Riksdag (Parliament) may delegate the power to enact certain rules to the Government, which in turn may sub-delegate powers to various public authorities. The Government has no power to intervene in an agency's decisions on specific matters relating to the application of the law or the decisions within its authority. Collective Government decision-making and the ban on instructing agencies on individual matters illustrate the prohibition of 'ministerial rule', as it is often called. The Riksdag is responsible for ensuring that ministerial rule does not occur. Should the Government be of the opinion that an agency has not applied a law correctly, its only remedy is to seek to amend the relevant legislation with a clarification [4]. During the first 2 years of the pandemic, the PHAS has, according to its commission, issued almost 100 Regulations and items of General Advice related to COVID-19.

The PHAS coordinated the national response to the pandemic in accordance with its mandate. This work involved not only a close dialogue with regions, municipalities and other Government agencies, but also with many private actors such as representatives of the hospitality industry, arts and culture, and organisers of major events. Furthermore, before a final decision, all regulations went, often on short notice, through an official review with stakeholders that would be affected by the proposed regulation.

## Measures to reduce the spread of COVID-19 in Sweden

Diagnostics for COVID-19 were available at the PHAS on 17 January 2020 and diagnostic capacity was established in the 21 regions during the subsequent weeks and months. In order to increase national testing capacity, collaboration with other actors with laboratory capacity was established, but the main capacity remained with laboratories associated with the regional authorities. Actors included the private sector as well as other national agencies and academia. Initiatives were taken during March 2020 and intensified when PHAS was tasked by the Government on 30 March 2020 to increase the testing nationally. As testing capacity grew, the testing strategy evolved from targeting prioritised groups to large-scale national testing of all individuals with symptoms by June 2020. Throughout the pandemic, testing to avoid severe disease, ensure correct medical treatment and prevent further transmission of the virus has been prioritised.

From late February 2020 onward, the PHAS issued recommendations to reduce the spread of COVID-19. Recommendations including staying at home even with very mild symptoms, minimising contact with people older than 70 years and other risk groups and, for people older than 70 years, taking extra care to avoid close contact with others. The measures were further developed and formally adopted as general (mandatory) guidelines on 1 April 2020. The general guidelines included advice to avoid close contact indoors and outdoors, avoid gatherings, work from home if possible and avoid unnecessary travel. These guidelines were in force during the 2-year pandemic period and were continually updated and adjusted in accordance with the development of the pandemic and growing knowledge about the virus and the disease. In February 2022, all measures were removed. Several indicators, for example the movement of people as observed by mobile phone data [5] and the incidence of other respiratory infections [6-8], were used to monitor the effect of the recommendations and supported that compliance was high. 

Government regulations were enacted to prohibit public gatherings starting on 12 March 2020. The permitted size of public gatherings varied over time; it started with a ban on gatherings of over 500 persons on 11 March [9], was soon thereafter, on 27 March, lowered to 50 [10] and later, all gatherings were essentially banned as a maximum of eight persons were permitted [11]. Specific and legally enforceable restrictions for serving establishments were issued on 24 March 2020 [12], and restrictions in these environments also varied over time. Similar regulations were enacted for other public establishments, for example fairs and amusement parks [9-11].

In addition to the legal acts described above, the PHAS developed a number of recommendations based on the knowledge gathered. These covered different aspects such as testing, contact tracing, and rules of conduct for individuals infected with COVID-19, as well as a range of different situations and environments, among them schools. For a complete summary of measures taken refer to the COVID-19 timeline available at PHAS [13]. On 9 February 2022, the majority of measures for the general population were discontinued based on the epidemiological development and the fact that the pressure on the hospitals was diminishing.

The most important measure was vaccination, which started on 28 December 2020 [14]. In the autumn of 2021, the entire population aged 18 years and older were offered a third dose and all children 12 years and older had been offered two doses. Among children younger than 12 years, only those in medical risk groups were offered vaccination. By 5 September 2021, 89% of those aged 60–69 years and 91% those aged 70 years and older had been vaccinated with two doses. By 8 May 2022, 86% of individuals 18 years and older had been vaccinated with two doses and 65% with three doses. However, of those aged 60–69 years and those aged 70 years and older, 84% and > 90%, respectively, had received three doses of vaccine.

**Figure 1:**
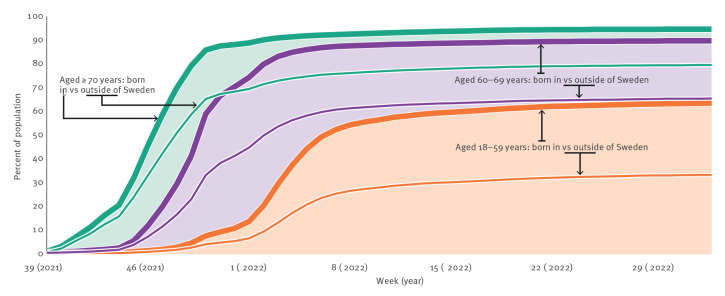
Cumulative vaccination coverage with three doses of COVID-19 vaccine, by origin and age group, Sweden, week 39, 2021–week 33, 2022

A major challenge since the start of the vaccination has been to reach the whole population. Despite considerable efforts by all involved stakeholders, uptake has been comparatively low among individuals born outside of Sweden, those in low income strata and individuals with low education levels. By May 2022, vaccination coverage for individuals aged 18–59 years who were born in Sweden was 88% and 62% for dose 2 and dose 3, respectively, while uptake among those born outside of Sweden only reached 67% and 32%. In those aged 60–69 and ≥ 70 years, vaccination coverage with three doses among Swedish-born individuals was 89% and 94%, respectively, compared with 65% and 79% for individuals born outside of Sweden (Figure 1). At the same time, vaccination coverage with dose 3 in those aged 30–64 years and in the lowest income quintile was 41% compared with 79% for the highest income quintile. For ages ≥ 65 years, this was 84% in the lowest income quintile and 95% in the highest income quintile. Similar trends were seen for education level [15].

Bold lines: individuals born in Sweden; thin lines: individuals born outside of Sweden.

The Swedish strategy was to limit the spread of COVID-19 while concurrently enabling classroom teaching for children up to 15 years of age, to support the physical and mental health of children and youth, and to reduce the loss of knowledge and negative long-term consequences on public health. A holistic public health perspective, the best interests of the child and the advantages of classroom teaching were consistently promoted through national guidelines and recommendations concerning online vs classroom teaching. The guiding principles of a long-term public health perspective, the principle of proportionality and the child’s best interest are stated in the Communicable Disease Act. As a result, children under the age of 16 years were largely able to receive classroom teaching throughout the pandemic [16,17].

Confidence in national agencies is high in Sweden [18,19]. Annual national surveys also show that Swedish citizens have high confidence in healthcare services and in the PHAS, and two out of three respondents reported that they have high confidence in how the PHAS has handled the pandemic [20,21]. Together with the National Board of Health and Welfare and the Swedish Civil Contingencies Agency, the PHAS held regular press conferences that included time for media questions and individual interviews with experts [22]. The COVID-19-related information on the PHAS’s website was continually updated with information for the public, those responsible for workplaces and healthcare professionals. Information was presented in formats tailored to specific target groups such as older people, children and pregnant women, those with hearing loss or vision impairment, and those for whom Swedish is not a first language or who lack knowledge of Swedish.

## Public health impact of the pandemic in Sweden

**Figure 2:**
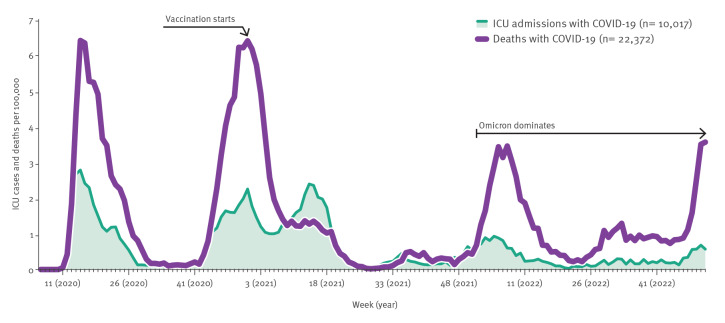
Number of new ICU admissions and deaths with laboratory-confirmed COVID-19 per 100,000 population per week, Sweden, February 2020–May 2022

During its first 3 years, the pandemic in Sweden had five waves of varying intensity (Figure 2). In 2020, there were two waves peaking in April and December, with many hospitalised patients and deaths. The highest weekly hospitalisation rate in the first and second wave was 18 new hospitalised patients with COVID-19 per 100,000 individuals. The spread of the Alpha variant of SARS-CoV-2 in early 2021, just as vaccination against COVID-19 was being rolled out, caused a third wave that peaked in April. The highest weekly hospitalisation rate during the third wave was 14 new patients per 100,000 individuals, but fewer deaths and fewer intensive care unit (ICU) admissions occurred during that time. The introduction of the Delta variant that caused a high peak in many countries did not have a great impact in Sweden. Instead, a fourth wave in Sweden started in in late 2021, partly due to the rapid spread of the Omicron variant.

ICU: intensive care unit.

Source: Data from the Swedish Intensive Care Registry and Sweden’s national database for notifiable diseases and the National Vaccine Register. Vaccinations against COVID-19 started in December 2020.

**Figure 3:**
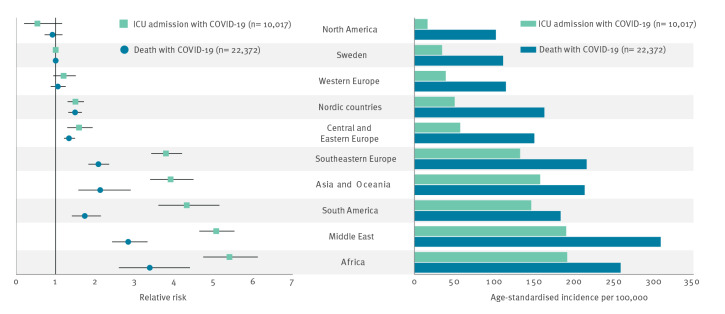
Relative risk and age-standardised incidence for ICU admissions and deaths, by region of birth, Sweden, March 2020–February 2021

In total, 9,816 individuals with laboratory-confirmed COVID-19 died within 30 days of diagnosis at an average age of 83 years (range: 0– ≥ 100) during 2020 and 5,534 at an average age of 80 years during 2021 (range: 0– ≥ 100). In 2022, 7,022 died, with an average age of 84 years (range: 0– ≥ 100). This corresponds to a rate of 95 deaths per 100,000 population in 2020, 53 in 2021 and 67 in 2022. The majority of deceased COVID-19 cases lived in care facilities for elderly people or had at-home care services (74% in 2020 and 54% in 2021). In 2020, 40 patients with laboratory-confirmed COVID-19 per 100,000 population were admitted to an intensive care unit, at an average age of 61 years (range: 0–97). In 2021, 39 patients per 100,000 population, with an average age of 60 years (range: 0–98), were admitted and in 2022, the figures were 17 per 100,000 and an average age of 60 years (range: 0–97). Of the patients in ICU with laboratory-confirmed COVID-19 during 2020 and 2021, 70% were male and during 2022, 61% were male. Of the deceased COVID-19 cases during the 3 years, 55% were male. During the first year of the pandemic, individuals who were born abroad had an increased risk both of requiring ICU care and of dying, compared with individuals born in Sweden. In some cases, their relative risk of ICU admission with COVID-19 was five times that of Swedish-born individuals (Figure 3). Similar trends were seen in regard to mortality, where for some groups, the risk of dying with COVID-19 was up to three times that of Swedish-born individuals. Further analysis has shown that a combination of socioeconomic risk factors, such as risk of poverty, low educational level and living in crowded conditions, explains the difference between individuals born in vs outside of Sweden [23].

ICU: intensive care unit.

Source: Data from the Swedish Intensive Care Registry and Sweden’s national database for notifiable diseases. Incidences were standardised with age- and sex-specific weighting (5-year categories). Relative risk was calculated with Poisson regression and adjusted for age and sex.

### Excess mortality and long-COVID

High excess mortality was seen in Sweden during the two waves of COVID-19 in 2020 and, to a lesser extent, during the Omicron wave in 2022. Excess mortality in Sweden was higher among men than among women but no excess mortality was seen during 2021 [24,25]. This followed the same pattern as the reported mortality. The differences between regions were greatest during the first wave when the Stockholm region was worst affected. As reported in *The Lancet* by the COVID-19 Excess Mortality Collaborators, the estimated excess mortality rate was 91 per 100,000 in Sweden compared with 140 per 100,000 in western Europe between 1 January 2020 and 31 December 2021 [26]. In the same period, Denmark had 94, Finland 80 and Norway 7 per 100,000 [26].

The number of persons with long COVID in Sweden is to a great extent unknown. The National Board of Health and Welfare is mandated with following the development and estimates that between October 2020 and October 2022, 30,000 persons were treated with the diagnosis in Sweden. In April 2023, more than 100 persons were treated each week according to its statistics [27].

### Mental health and well-being

In the Swedish National Public Health Survey [28], a number of indicators are used to monitor mental health. For non-specific psychological distress in the general population, as measured by the Kessler 6 screening scale [29], we so far lack sufficient data to determine if there have been any changes in connection with the COVID-19 pandemic. When it comes to self-reported anxiety among 16–84-year-olds, there has been an increase from 41% in 2020 to 43% in 2021 [30]. However, the increase is in line with the trend before the pandemic, and so a connection cannot be established.

Despite worries that substance abuse and gambling problems would increase during the pandemic, a trend analysis (personal communication Jessika Spångberg, January 2023) based on the National Public Health Survey shows a slight decline in the prevalence of past-year use of cannabis among 16–84-year-olds in 2021 compared with previous years. For other narcotic drugs, no significant changes were observed. Furthermore, there has been a small decline in the prevalence of self-reported harmful alcohol use during the pandemic. The decreasing trend in the proportion reporting daily smoking during the period 2006 to 2020 continued in 2021. Data from the Swedish Longitudinal Gambling Study shows that, in total, gambling problems have not changed significantly during the pandemic. However, the prevalence of gambling problems among unemployed people has increased [31].

In the 2021 National Public Health Survey, 67% of the Swedish population reported being physically active for at least 150 min per week [32]. In total, there has been a small increase since 2016 in the proportion of 16–84-year-olds reporting recommended levels of physical activity, with no apparent changes during the pandemic.

The survey also shows that there have been changes in health indicators among 16–29-year-olds, but they are generally in line with trends already identified before the pandemic. That is, mental health among the young has been slowly deteriorating over several years, and that trend has continued. Self-reported smoking and harmful alcohol use has continued to decrease, and the proportion reaching recommended levels of physical activity has remained unchanged. However, the previous trend with an increasing proportion of 16–29-year-olds eating at least two servings of vegetables per day did not continue in 2021. In addition, qualitative interviews among 16–29-year-olds living under challenging conditions (socially, economically or health-related), indicate that the pandemic has interacted with the previous life situation and in some cases reinforced pre-existing challenges [33].

## Conclusion

Pandemic management needs to take into account the country context, as measures used in one country might not be effective in another or even possible to implement. The Swedish response has been closely tailored to our national context and continually modified to the development of the pandemic. It involved close collaboration with many stakeholders in society. We believe that similar results were achieved as in other countries in terms of minimising physical contacts and restricting the spread of the virus. This is supported by mobile phone movement data and a very low incidence of influenza and other respiratory infections. Morbidity and excess mortality during the first 3 years of the pandemic have also been similar in Sweden as in most other western European countries. This is verified by published comparisons of excess mortality as well as data from the European Centre for Disease Prevention and Control. The consequences on mental health and healthy living behaviours that are measured in Sweden have, so far, been limited to a small proportion of the population. Notably, despite worries of increased drug use, there has been a small decrease in both self-reported harmful alcohol use and past-year cannabis use during the pandemic. However, some effects may take more time before they become apparent, and some groups have been more affected than others.

Even though the Swedish approach managed to flatten the epidemic curve, reduce the spread of the disease and achieve a high level of vaccination, it was less successful in protecting vulnerable groups. Especially during the first and the second wave, older people were severely affected, especially those living in long-term care facilities. Furthermore, individuals born outside of Sweden, in low income strata and with low education levels had a higher level of mortality and morbidity.

Finally, in Sweden, trust in public institutions is high, which has been important and is part of the context that makes it possible to manage a pandemic with broad public health measures that allow society to continue to function as normally as possible. The adherence to recommendations has been very high in Sweden which is a result of the trust that has been established since long before the pandemic. It is important to maintain this trust in order to be able to handle other crises in the future and tackle long-term societal challenges.

There are a number of areas where Sweden needs to improve its pandemic preparedness both in the health sector and in other parts of society. It is important that we make use of the experiences from this pandemic to develop and improve those areas further.
